# Human epidermis models demonstrate mediator role of TLR2 and TLR3 for psoriatic inflammation

**DOI:** 10.3389/fmed.2025.1663279

**Published:** 2025-09-09

**Authors:** Hanna Glasebach, Lukas Denzinger, Steffen Rupp, Anke Burger-Kentischer

**Affiliations:** Department Cell and Tissue Technologies, Fraunhofer Institute for Interfacial Engineering and Biotechnology IGB, Stuttgart, Germany

**Keywords:** psoriasis, Toll-like receptor, *in vitro* epidermis model, keratinocytes, cytokines

## Abstract

Psoriasis is a multifactorial systemic autoinflammatory disease that is characterized by complex signaling between keratinocytes and immune cells. The trigger factors for the cutaneous manifestation of the disease are divers but have in common that they induce an activation of the Toll-like receptor (TLR) signaling pathways. This is best described for the activation of TLR7/8/9 in dendritic cells. In this study, we investigated the role of TLR2 and TLR3 activation in keratinocytes for the expression of psoriatic hallmarks in the skin. We set up 3D epidermis models using wild type keratinocytes and TLR2 knockout and TLR3 knockout (KO) keratinocytes derived from the wild type keratinocytes and treated them with TLR agonists. Immunohistochemical, western blot, and multiplex analysis showed that the TLR activation induced the expression of psoriasis associated markers like S100A7, p-STAT3, CXCL-1, IL-8, IL-1α, S100A9, and IL-23 in the wild type but not in the TLR KO epidermis models. Thus, TLR2 and TLR3 activation in keratinocytes individually contributes significantly to inducing the release of cytokines and other immune modulators characteristic for a psoriasis like inflammation in 3D epidermis models.

## 1 Introduction

Psoriasis is a chronic inflammatory lifelong disease of the skin with an incidence of about 2% in western countries. It is caused by a dysregulated immune defense of the skin leading to inflammation and aberrant proliferation of keratinocytes.

Psoriasis is a multifactorial disease with a complex genetic background and involvement of a wide range of different cell types from immune cells to keratinocytes and other skin-resident cells. The interaction of the different cell types with each other is crucial for the disease pathogenesis. However, our understanding of the distinct roles of each of these cell types is still incomplete (1). The research in the last 20 years was mainly focused on immune cells, however, keratinocytes are recently gaining more attention (2, 3). For simplification, psoriasis pathogenesis is often divided in two phases: the initiation and the maintenance of the disease. Keratinocytes play a role in both of them. In the initiation phase of psoriasis, cellular stress causes keratinocytes to release danger associated molecular patterns (DAMPs), like self-DNA and antimicrobial peptides which activate skin resident dendritic cells (4, 5). Further, keratinocytes respond to cytokines from immune cells with the release of more cytokines and chemokines and, thus, contribute to the inflammatory surrounding and the maintenance of the disease in form of a positive feedback loop (6). This can be mimicked *in vitro* using typical epidermal skin models (7).

Psoriatic inflammation is triggered in genetic predisposed individuals by various external factors ranging from skin injury to infection and sun exposure. One common attribute of all of these factors is the activation of pathogen recognition receptors (PRR) such as Toll-like receptors (TLR) (8). TLR are innate immune receptors that are crucial for the first line of host defense but are also related to immune disorders. The activation of TLR7, 8, 9 on dendritic cells by endogenous danger signals is well described as an initiating event for skin inflammation in psoriasis (9). The key role of the endosomal TLRs is further supported by a widely used mouse model, in which imiquimod, a TLR7 agonist, is sufficient to induce a psoriatic skin reaction (10). Further several TLR7, 8, 9 antagonists were tested in clinical trials and showed promising results (11, 12). In contrast to that, the role of TLR on other skin cell types, like keratinocytes, and their involvement in the initiation phase of psoriasis is less well studied.

Alterations in the expression of TLR in psoriatic keratinocytes have been observed previously. In psoriatic lesions, TLR3 and TLR2 are expressed at a higher level in the upper epidermal layers compared to normal skin (13–15).

TLR3 is located in the endosome and one of the key receptors for the innate antiviral immunity. TLR3 is activated by double stranded RNA (dsRNA) from exogenous origin upon viral infections or from endogenous origin after tissue damage (4). TLR3 mediates an antiviral response in keratinocytes and promotes tissue reepithelization (16, 17). Dysregulated TLR3 activation can lead to chronic inflammation and is linked to various autoimmune diseases (18).

In psoriasis patients an increased amount of self-RNA released from necrotic keratinocytes was found in the blood, in the skin and associated to neutrophil extracellular traps (NET) (19, 20). It was postulated that the combination of free RNA and the antimicrobial peptide LL37 promotes an inflammatory response in keratinocytes and contributes to psoriatic plaque formation (21). One relevant receptor for RNA recognition is TLR3. The activation of TLR3 in keratinocytes by dsRNA induces the expression of IL-23, S100A7, and IFN-ß (5, 20). Further, the interaction of keratinocytes with NETs containing RNA was shown to stimulate human beta defensin 2 (hBD2) expression (22). All of these factors are associated with psoriasis.

TLR2, located on the cell surface, recognizes a wide range of microbial ligands and mediates the innate immune response to bacterial infections. However, TLR2 can also be activated by DAMPs like the psoriasis associated antimicrobial peptide S100A9 (23, 24). In a murine psoriasis model, TLR2 signaling contributed significantly to the itching by mediating the expression of CXCL1/2, IL-31, IL-33, ST2, IL-6, and TNF-*α* (25). Further, two different TLR2 gene polymorphism, detected in a Turkish and a Chinese population, were associated with psoriasis vulgaris (26, 27).

In this work, we strived to shed more light on the role of TLR2 and TLR3 in keratinocytes for psoriasis and evaluate both receptors as a potential target for psoriasis therapies. For this, we knocked-out either TLR2 or TLR3 in keratinocytes and used the cells for the set-up of organotypic 3D *in vitro* epidermal equivalents. We stimulated the epidermis models with TLR agonists and analyzed cytokine profiles and the expression of psoriasis markers in comparison to epidermis models set up from unmodified keratinocytes to identify TLR mediated psoriatic effects.

## 2 Methods

### 2.1 Cell culture

The human keratinocyte cell line Ker-CT (ATCC, CRL-4048) was cultivated in DermaLife K keratinocyte growth medium (CellSystems, LM-0027) with 1% penicillin/streptomycin.

### 2.2 Generation of TLR KO keratinocytes

CRISPR/Cas9 mediated knock-out of TLR2 and TLR3 in keratinocytes was carried out by lipofection of Cas9 protein and sgRNAs with the Lipofectamine™ CRISPRMAX™ Cas9 Transfection Reagent (Invitrogen™) according to the manufacturer’s instructions. Briefly, 1.2 μg TrueGuide™ Synthetic sgRNA (Invitrogen™) and 6.25 μg TrueCut™ Cas9 Protein v2 (Invitrogen™) were combined with the transfection reagent complex, incubated for 10 min at room temperature to form ribonucleoproteins and added dropwise to adherent keratinocytes in 6-well plates. For the TLR2 knock-out the TrueGuide™ Synthetic sgRNA CRISPR811733_SGM targeting TLR2 exon 3 (sequence 5´-GACCGCAATGGTATCTGCAA-3′), for TLR3 knock-out the TrueGuide™ Synthetic sgRNA CRISPR1028685_SGM (sgRNA 1, sequence 5′-GCCTTGTATCTACTTTTGGG-3′) and CRISPR1028655_SGM (sgRNA 2, sequence 5´-TACCAGCCGCCAACTTCACA-3′) both targeting TLR3 exon 2 were used. The transfected keratinocytes were cultured for 48 h before they were passaged and expanded.

### 2.3 FACS analysis of TLR2 KO in keratinocytes

The cells were harvested and filtered through a 30 μm cell strainer. For staining of TLR2, 1×10^6^ cells were resuspended in 100 μL DermaLife K keratinocyte growth medium with 1% FCS, 2 μL of fluorescence labeled antibody [anti-TLR2-PE (Miltenyi, ref. no. 130–127-922)] or the corresponding isotype control antibody [REA control antibody IgG (Miltenyi, ref. no. 130–104-613)] was added and samples were incubated for 30 min at 37°C, 5% CO_2_ in the dark. The cells were washed twice with staining buffer and analyzed with a SH800S cell sorter (Sony). A two-way sort was used to separate TLR2 positive and negative keratinocytes.

### 2.4 Generation of epidermal equivalents

Epidermal equivalents were set-up as previously described with minor modifications (7). Keratinocytes (0.15 × 10^6^ cells/insert) were seeded in 0.4 μm Nunc trans-well cell culture inserts and cultured submerse in DermaLife K keratinocyte submerse medium (DermaLife K keratinocyte growth medium supplemented with 1.86 mM CaCl_2_). After 3 days, the medium was changed and the keratinocytes were cultured submers in DermaLife K keratinocyte differentiation medium (DermaLife K keratinocyte growth medium supplemented with 1.86 mM CaCl_2_ and 50 μg/mL ascorbic acid) for 24 h. Subsequently, the models were raised to the air-liquid interface by adding differentiation medium outside the insert and exposing the epidermis surface to the air. The medium was changed every 2–3 days. At day 5 of the airlift culture, specific TLR agonists [Pam_2_CSK_4_ (InvivoGen, tlrl-pm2s-1), poly(I:C) (InvivoGen, tlrl-pic)] were added to the cell culture medium. At day 11, the constructs were subjected to immunohistochemical staining. Samples of the cell culture supernatant were stored at −80°C for analysis of soluble immune mediators.

### 2.5 Western blot analysis

At the end of the experiment, epidermis models were cut out of the inserts and stored at −80°C. For the western blot analysis, the models were lysed in 150 μL RIPA buffer with 1x HALT™ protease inhibitor cocktail (Thermo Scientific™) on ice for 30 min. The protein concentration in the cell lysates was determined by a BCA assay. 25–30 μg of denatured protein per sample was separated by SDS gel electrophoresis in a NuPage® Novex® 4–12% Bis-Tris protein gradient gel (Invitrogen™). Separated proteins were transferred on a nitrocellulose membrane by semi-dry blotting at 20 V for 7 min. The membrane was blocked in 5% milk powder in TBS-T and incubated with the primary antibody in 5% milk powder/TBS-T or 5% BSA/TBS-T over night at 4°C. The following primary antibodies were used: anti-S100A7 (Novus Biologicals, cat no NB100-56559, 1:500), anti-phospho-STAT3 (Abcam, cat no ab76315, 1:1000), anti-STAT3 (Abcam, cat no ab119352, 1:1000). *β*-Actin (cell signaling technology, cat no 4970S, 1:5000) was used as loading control. The next day, the secondary horseradish peroxidase (HRP) coupled antibody (anti-rabbit from Sigma-Aldrich, cat no A0545 and anti-mouse from Jackson Immuno Research, cat no 115–035-068) was applied in 5% milk powder/TBS-T for 1 h at room temperature and afterwards the membrane was developed with Pierce™ ECL western blot substrate.

The intensity of the western blot bands was quantified using the software ImageJ from the National Institutes of Health (NIH) (28) and normalized to actin. The protein expression of TLR3, p-STAT3, and S100A7 in the different experimental conditions is shown as fold changes relative to the untreated wild type epidermis models in box plots. Mann–Whitney tests were performed with the software Origin to determine statistically significant differences between the distributions of the different models and experimental conditions.

### 2.6 Histology and immunohistochemistry

Histological and immunohistochemical stainings were performed as described in Glasebach et al. (7). Briefly, tissue sections were fixed in Bouins solution (Carl Roth GmbH), embedded in paraffin with a routine program and tissue slices were prepared. Tissue slices were either stained with hematoxylin (Merck KGaA) and eosin (Sigma-Aldrich) or immunohistochemically with specific antibodies. Therefore, masked epitopes were recovered by heat-induced antigen retrieval, followed by blocking with 5% BSA for 30 min and incubation with the primary antibody [anti-S100A7 (BioTechne Sales Corp., cat no NB100-56559, 1:500)] in Dako antibody diluent (Agilent Technologies, cat no S2022) at 4°C overnight. The biotin coupled secondary antibody (BioGenex, cat no LP000-ULE) in link diluent (1:100) and the streptavidin linked peroxidase in label diluent (1:100) were each applied for 30 min at room temperature. Between each step, the tissue sections were washed thrice in TBS-T. 3-Amino-9-Ethylcarbazole (AEC) chromogen diluted in peroxidase buffer (BioGenex, HK092-5 K) was used for visualization of the protein expression. The tissue sections were counterstained with hematoxylin and mounted with Aquatex solution.

### 2.7 Cytokine/chemokine multiplex assay

After stimulation of the epidermis models with TLR agonists, the supernatants were analyzed with a customized human Luminex® discovery assay (Bio-Techne, cat no LXSAHM) for the analytes TNF-*α*, IL-6, IL-8, S100A8, IL-10, IL-7, IL-1α, CXCL-5, G-CSF, IL-17C, PDGF-AA, IL-15, IL-12, CXCL-1, IL-18, IL-23, S100A9, IL-36b. The samples were used without further dilution and the assay was performed according to the manufacturer’s instructions. The readouts were obtained with a Luminex® xMAP® Intelliflex system and the results were analyzed using the Quantist™ software. Results are presented as boxplots. For statistical analysis, non-parametric Mann–Whitney tests were performed with the software Origin (OriginLab). The following comparison were made: the difference in analyte secretion between untreated and Pam_2_CSK_4_ treated wild type models was compared to the difference in analyte expression between untreated and Pam_2_CSK_4_ treated TLR2 KO models, the analyte levels of untreated wild type models were compared to the levels of poly(I:C) stimulated wild type models and to untreated TLR3 KO models, and the measured analytes in untreated TLR3 KO models were compared to poly(I:C) stimulated TLR3 KO models. The calculated *p*-value is indicated in the figures: *p < 0.05, **p < 0.01.

## 3 Results

### 3.1 Stimulation of *in vitro* epidermis models with TLR2 or TLR3 agonists

To analyze the role of TLR2 and TLR3 in psoriasis, we used 3D *in vitro* epidermis models for all experiments which mimic the complex stratification of the human skin. The 3D epidermis models were differentiated from primary immortalized keratinocytes and cultured under air-liquid interphase conditions. The physiological expression of epidermal markers in the *in vitro* models was shown in previous work (29). To examine the effect of TLR activation on keratinocytes, the epidermal models were either treated with the TLR2 agonist Pam_2_CSK_4_ or the TLR3 agonist poly(I:C) and subsequently analyzed for the expression of S100A7, an antimicrobial peptide and prominent psoriasis marker, by immunohistochemical staining and western blot.

**Figure 1:**
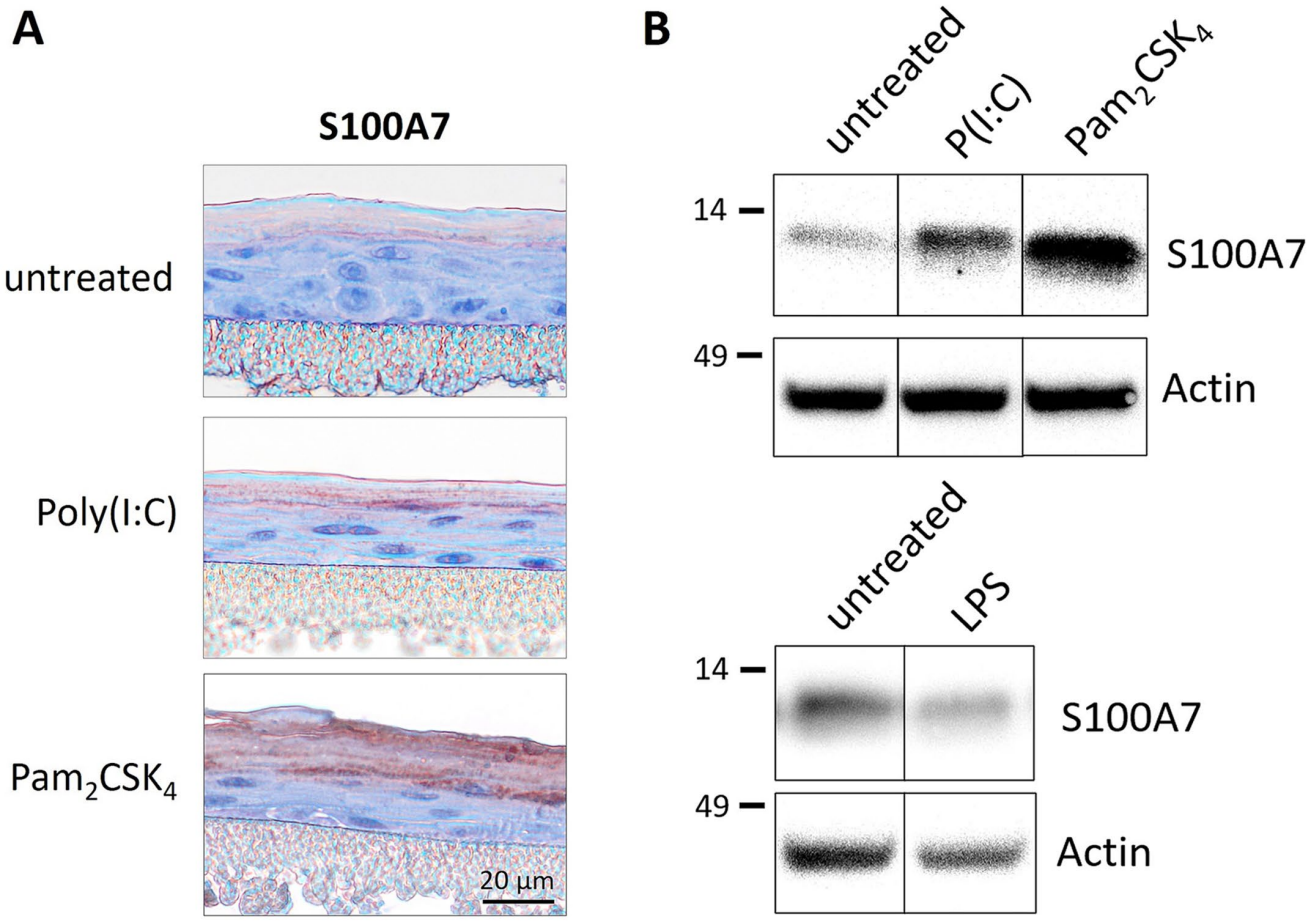
Treatmentof 3D epidermis models with TLR agonists. Epidermis models were set up from primary immortalized keratinocytes and stimulated with TLR2 agonist Pam_2_CSK_4_ (500 ng/mL), TLR3 agonist poly(I:C) (5 μg/mL) or TLR4 agonist LPS (0.8 ng/mL) for 6 days. The epidermis models were analyzed for the expression of S100A7 either immunohistochemically **(A)** or by western blot **(B)**. The protein molecular weight on western blot is indicated in kDa.

S100A7 is weakly expressed in the *stratum corneum* of unmodified epidermis models. The treatment with the TLR agonist poly(I:C) or Pam_2_CSK_4_ strongly increased the S100A7 expression in the cornified layer as shown in immunohistochemical staining (Figure 1A). This was confirmed by western blot analysis. Epidermis models stimulated with the TLR2 or TLR3 agonist exhibited enhanced protein levels of S100A7 (Figure 1B, upper panel). To validate the specificity of the observed effects, the TLR4 agonist LPS, a very strong endotoxin, was applied on the models. TLR4 stimulation did not increase S100A7 in the epidermis models as demonstrated in the western blot (Figure 1B, lower panel).

### 3.2 TLR2 and TLR3 KO epidermis models were stimulated with the respective agonist

**Figure 2:**
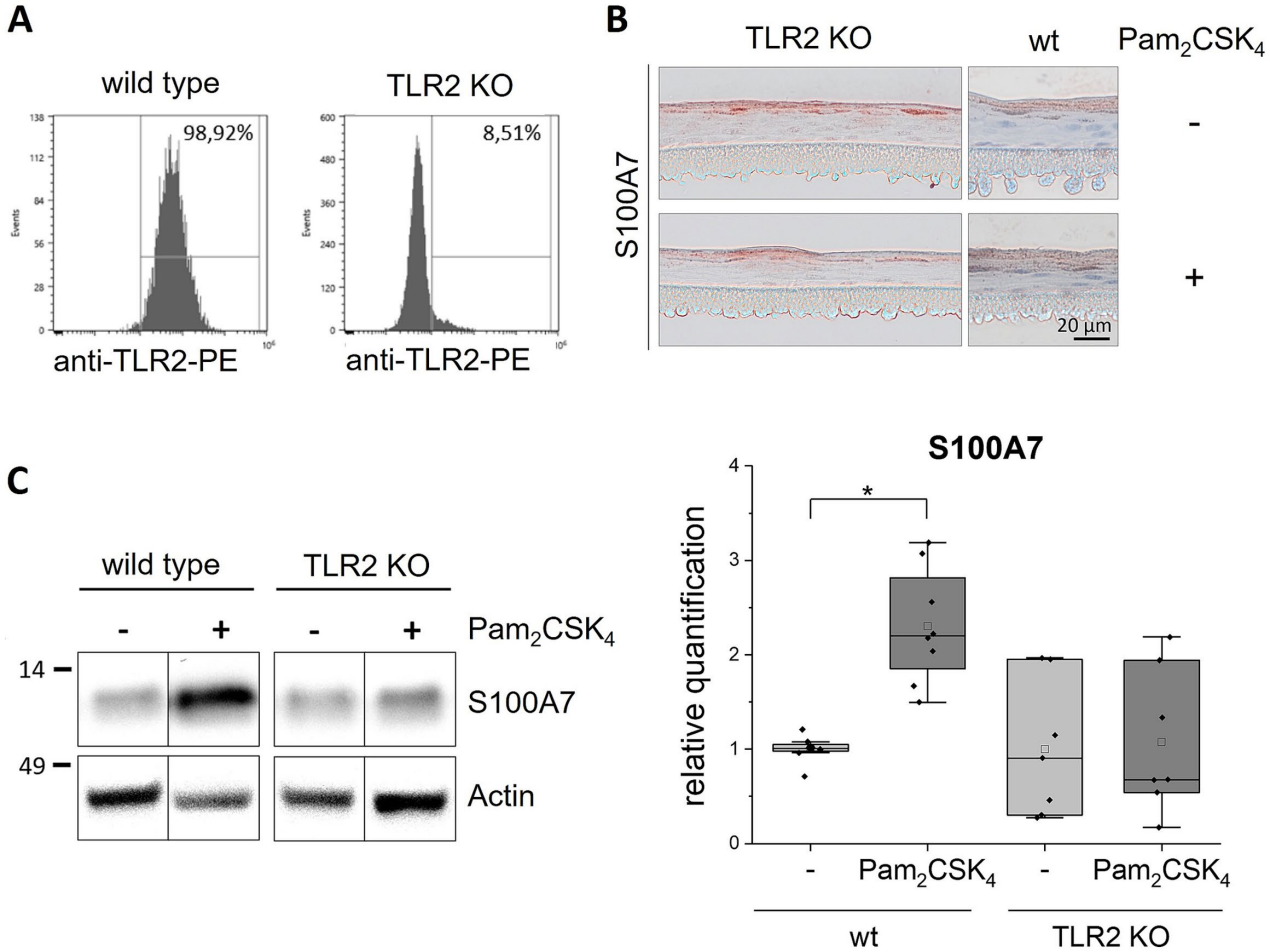
Stimulation of TLR2 KO epidermis models with Pam_2_CSK_4_. 3D *in vitro* epidermis models were set up from wild type or TLR2 KO keratinocytes and treated with 500 ng/mL Pam_2_CSK_4_ for 6 days during airlift culture. **(A)** FACS analysis of TLR2 expression (plasma membrane) in wild type and TLR2 KO keratinocytes. **(B)** Immunohistochemical staining of S100A7 in TLR2 KO and wild type (wt) epidermis models with and without Pam_2_CSK_4_ treatment. Representative images are shown for the visualization of the staining pattern. **(C)** Western blot analysis of S100A7 in wild type and TLR2 KO epidermis models (*N* = 2, *n* = 3). Full length blots can be found in the supplement (Supplementary Figure 2). Protein levels were normalized to actin and are shown as fold changes relative to the untreated wild type models in a box plot. The boxes show the interquartile range (IQR) with the median as horizontal line and the mean as unfilled square. Whiskers extend to the most extreme values within 1.5 × IQR from the quartiles, points beyond are plotted as outliers. * *p* < 0.05 (Mann–Whitney test).

**Figure 3:**
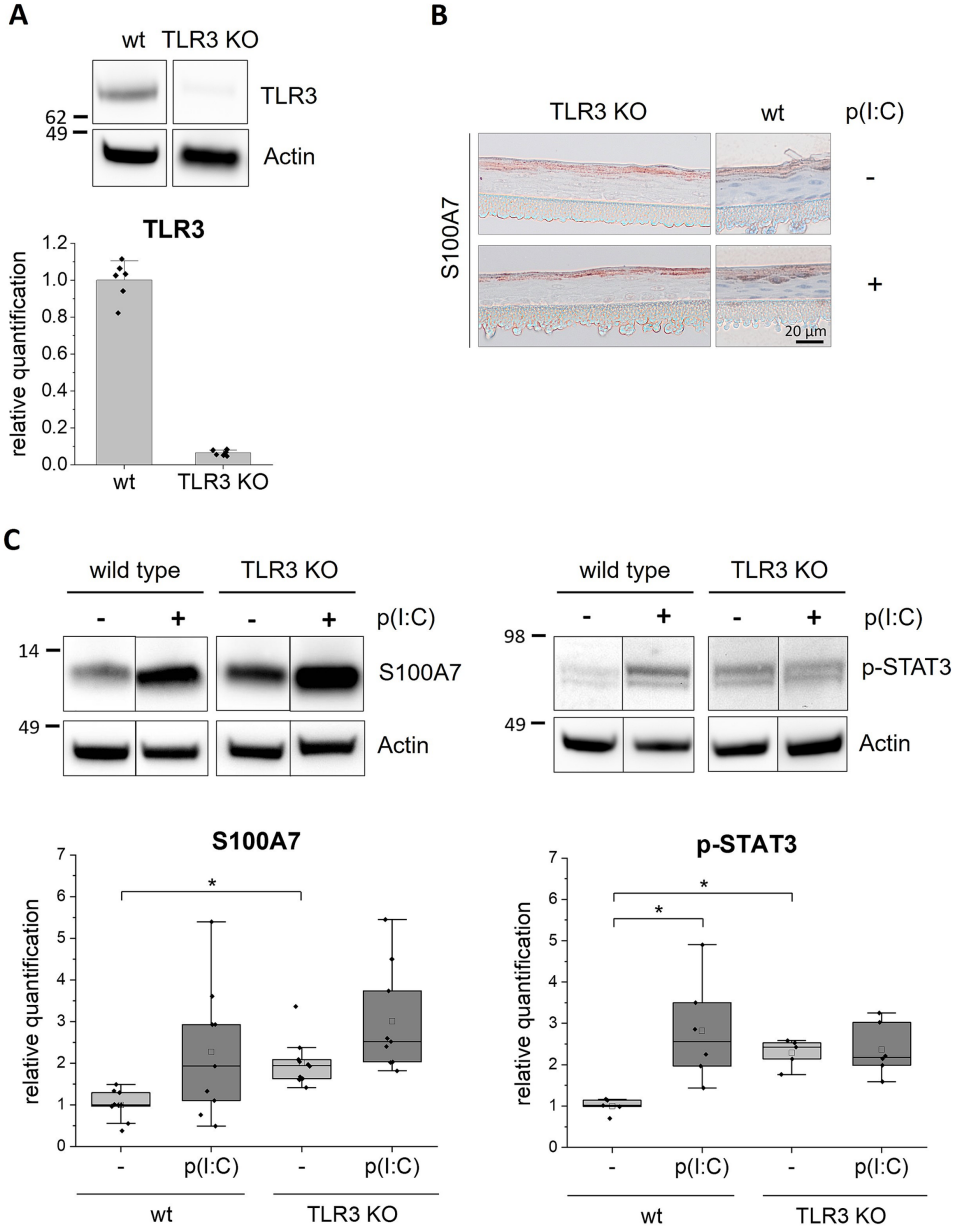
Stimulationof TLR3 KO epidermis models with poly(I:C). 3D *in vitro* epidermis models were set up from wild type or TLR3 KO keratinocytes and treated with 5 μg/mL poly(I:C) for 6 days during airlift culture. **(A)** Western blot analysis of TLR3 expression (endosomal) in wild type and TLR3 KO epidermis models. **(B)** Immunohistochemical staining of S100A7 in TLR3 KO and wild type (wt) epidermis models with and without poly(I:C) treatment. Representative images are shown for the visualization of the staining pattern. **(C)** Western blot analysis of S100A7 (*N* = 3, *n* = 3) and phosphorylated (Y705) STAT3 (*N* = 2, *n* = 3) in wild type and TLR3 KO epidermis models. Protein molecular weight is indicated in kDa. Full length blots can be found in the supplement (Supplementary Figure 3). Protein levels were normalized to actin and are shown as fold changes relative to the untreated wild type models in a box plot. The boxes in the plot show the interquartile range (IQR) with the median as horizontal line and the mean as unfilled square. Whiskers extend to the most extreme values within 1.5 × IQR from the quartiles, points beyond are plotted as outliers. * *p* < 0.05 (Mann–Whitney test).

Next, we established TLR2 and TLR3 knockout (KO) keratinocyte cell lines with the CRISPR/Cas9 method. The knockout efficiency was more than 90% as determined by FACS for the TLR2 KO (Figure 2A) and by western blot analysis for the TLR3 KO cells (Figure 3A). We set-up epidermis models from the TLR2 and the TLR3 keratinocytes and treated them with Pam_2_CSK_4_ and poly(I:C) respectively.

Stimulation of the normal epidermis models with Pam_2_CSK_4_ induced a significantly higher expression of S100A7 compared to the untreated control models. In contrast, the TLR2 KO models did not show any effect of the TLR2 agonist on S100A7 expression (Figure 2C). Non-quantitative immunohistochemical staining of the TLR2 KO epidermis models revealed that S100A7 was like in the wild type epidermis models localized in the *stratum corneum* (Figure 2B). Neither the intensity nor the distribution of the S100A7 staining changed in the TLR2 KO models after stimulation with the TLR2 agonist.

The TLR3 KO epidermis models were examined as described above for the TLR2 KO models.

The western blot analysis showed that the S100A7 expression level was about 2.5-fold enhanced after poly(I:C) treatment in normal epidermis models (Figure 3C, left panel). In the TLR3 KO epidermis models, S100A7 was about two times stronger expressed without any treatment compared to the wild type control. Furthermore, it increased about 1.5-fold after stimulation with poly(I:C). However, the increase in S100A7 expression upon poly(I:C) treatment was in the TLR3 KO models clearly less than observed for the wild type. Further, the stimulation with poly(I:C) did not affect the localization of S100A7 in the TLR3 KO models as shown in representative non-quantitative immunohistochemical stainings of the TLR3 KO epidermis models (Figure 3B). We also examined the activated phosphorylated form of the transcription factor STAT3 (p-STAT3), which is upregulated in psoriatic lesions. The western blot revealed that p-STAT3 was significantly increased in response to TLR3 stimulation with poly(I:C) in normal epidermis models whereas this was not seen for the TLR3 KO epidermis models (Figure 3C, right panel). Curiously, the p-STAT3 protein level was about two times higher in the TLR3 KO epidermis models compared to the untreated wild type epidermis models. Thus, deletion of TLR3 in keratinocytes resulted in a higher basal p-STAT3 level.

In TLR2 and TLR3 KO epidermis models the treatment with TLR2 or TLR3 agonists did not induce the expression of psoriasis markers. However, the KO of TLR3 in keratinocytes resulted in an overall higher expression of S100A7 and higher levels of activated STAT3 in the epidermis models. To verify these results, we generated an independent TLR3 deletion cell line using a second sgRNA 2 resulting in a frame shift at a different position on TLR3 exon 2 (Chr.4: 186076816–186,076,838 on GRCh38). This second cell line showed the same result, confirming the high expression of S100A7 in the untreated TLR3 KO epidermis models (Supplementary Figure 1).

### 3.3 Analysis of the secreted immune mediators after TLR stimulation in epidermis models

**Figure 4:**
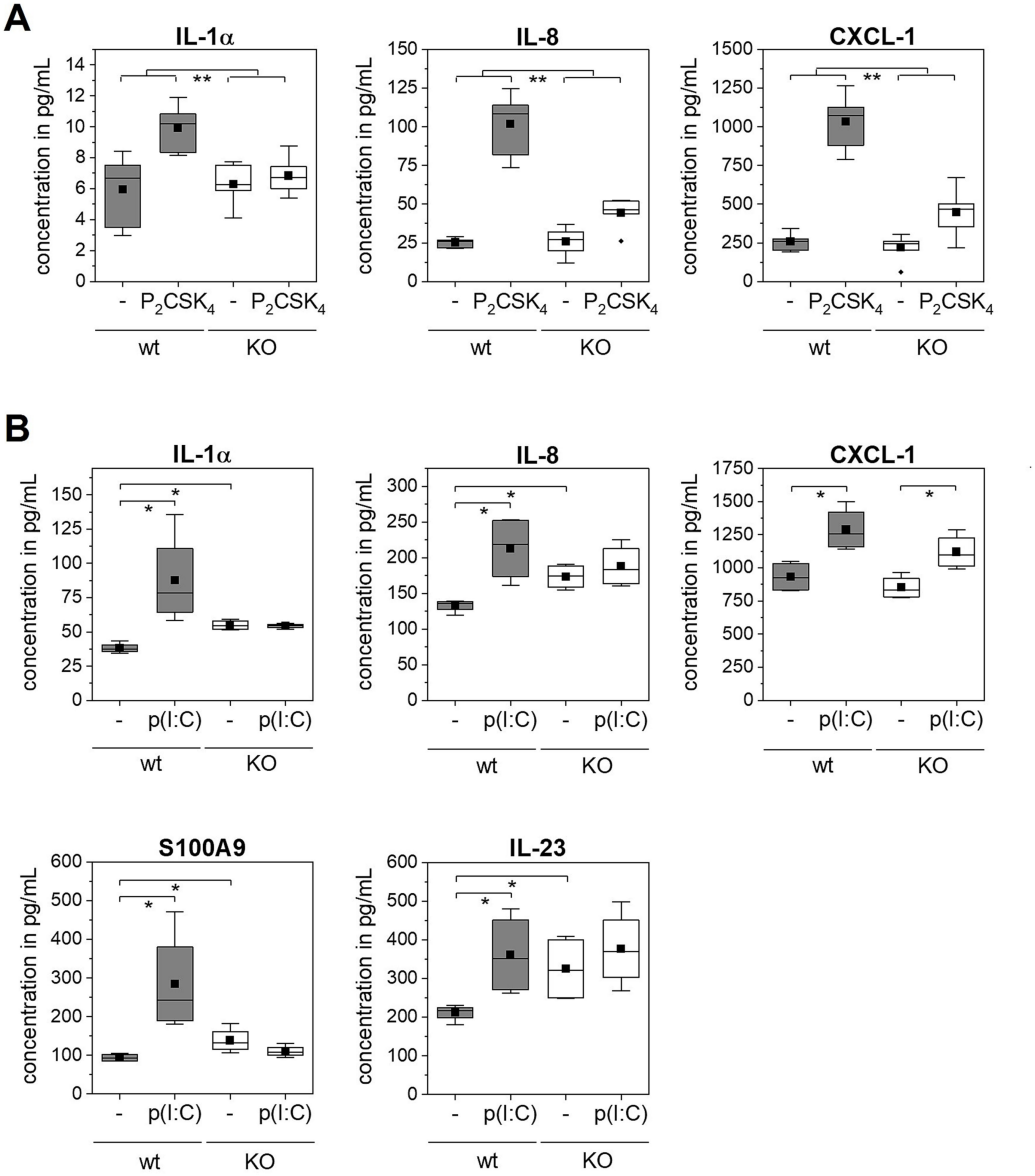
Multiplexanalysis of secreted immune mediators after TLR stimulation. 3D *in vitro* epidermis models were set up from wild type or TLR2 or TLR3 KO keratinocytes and treated with 5 μg/mL poly(I:C) or 500 ng/mL Pam_2_CSK_4_ for 6 days during airlift culture (*N* = 2, *n* = 4). Data is shown as box plots. The boxes indicate the interquartile range (IQR) with the median as horizontal line and the mean as black square. Whiskers extend to the most extreme values within 1.5 × IQR from the quartiles, points beyond are plotted as outliers. *p*-value (* *p* < 0.05, ** *p* < 0.01) was determined with Mann–Whitney test. **(A)** Comparison of wild type (gray boxes) and TLR2 KO epidermis models (white boxes) with and without Pam_2_CSK_4_ treatment (indicated as - / P_2_CSK_4_). **(B)** Comparison of wild type (gray boxes) and TLR3 KO epidermis models (white boxes) with and without poly(I:C) treatment (indicated as - / p(I:C)).

In addition to S100A7 and p-STAT3, the expression profiles of further psoriasis associated secreted immune mediators of the wild type and TLR2 or TLR3 KO epidermis models after stimulation with Pam_2_CSK_4_ or poly(I:C) were investigated with a multiplex assay (Figure 4). For six of all 18 investigated analytes (TNF-α, IL-6, IL-8, S100A8, IL-10, IL-7, IL-1α, CXCL-5, G-CSF, IL-17C, PDGF-AA, IL-15, IL-12, CXCL-1, IL-18, IL-23, S100A, IL-36b) significant differences were measured after stimulation with the agonists.

The treatment of the normal epidermis models with Pam_2_CSK_4_ induced the expression of the cytokine IL-1α and the chemokines IL-8 and CXCL-1 (Figure 4A). The measured IL-8 and CXCL-1 level in the supernatant was five to six times higher in epidermis models stimulated with the TLR2 agonist compared to the control models. In TLR2 KO epidermis models also a weak increase in all analytes was observed upon Pam_2_CSK_4_ treatment. This increase was, however, significantly lower compared to the effect of Pam_2_CSK_4_ on the normal epidermis models.

Normal epidermis models stimulated with poly(I:C) exhibited elevated levels of IL-1α, IL-8, CXCL-1, S100A9 and IL-23 compared to the control condition (Figure 4B). In contrast, we measured only a very weak or no response of the TLR3 KO epidermis models to the poly(I:C) treatment as none of the five mediators was significantly increased, except for CXCL-1. However, similar as we observed for S100A7, the TLR3 KO epidermis models showed in the untreated condition a significantly higher expression of IL-1α, IL-8, IL-23, and S100A9 compared to the wild type.

## 4 Discussion

Psoriasis is highly complex with multiple intrinsic and extrinsic contributing factors and involvement of a wide range of immune and skin cells. It remains a challenge to clarify the role of the different cellular players in initiation, maintenance and relapse of the disease. In this work we used a simple yet *in vivo* close normal epidermis model to focus specifically on the activation of TLR2 and TLR3 in keratinocytes.

S100A7, also called psoriasin, is expressed only at low levels in the skin of healthy individuals, but strongly upregulated in psoriatic lesions (30). We demonstrated that treatment of *in vitro* epidermis models with the TLR2 agonist Pam_2_CSK_4_ and the TLR3 agonist poly(I:C) induced the expression of the psoriasis marker S100A7 (Figure 1).

Further, we confirmed that S100A7 expression is specifically mediated by TLR2 activation with TLR2 KO epidermis models (Figure 2). These initial results were pointing toward a connection between TLR2 activation in keratinocytes and psoriasiform skin inflammation. Therefore, we examined more closely which other immune mediators were upregulated in the wild type but not the TLR2 KO epidermis models upon Pam_2_CSK_4_ treatment. In these experiments, we identified IL-1α and the chemokines CXCL-1 and IL-8 (Figure 4).

These three cytokines have been described in the chemotactic recruitment of neutrophils.

IL-1α is an alarmin, that is constitutively expressed by keratinocytes and released within others by necrotic cells in psoriatic plaques (31). A high abundance of IL-1α was observed in psoriatic lesions and overexpression of IL-1α in mice skin was sufficient to induce a psoriasis like phenotype (32). One of the effects of IL-1α described in the literature is that it acts on keratinocytes in an autocrine fashion and mediates the expression of chemokines like CXCL-1 and IL-8 (31, 33). Considering IL-1α as an enhancer of the immune response could explain why we measured only a small increase in IL-1α but a strong increase in CXCL-1 and IL-8 levels after stimulation with Pam_2_CSK_4_. However, as we focused on a time frame of several days and did not measure cytokine levels within the first hours after TLR stimulation, we cannot exclude that the chemokine expression was induced by the TLR agonist and not as a secondary effect by IL-1α.

The chemokines CXCL-1 and IL-8 are overexpressed in psoriatic skin compared to healthy skin (34). CXCL-1 and IL-8 are crucial for the chemotaxis of neutrophils to the skin (35). The accumulation of neutrophils in the epidermis of psoriatic patients was first described by Munro in 1898 as a hallmark of psoriasis and thereafter named Munros microabcesses (36). Neutrophils contribute significantly to the disease pathogenesis. Activated neutrophils cause a highly immunogenic environment in the skin by the release of radical oxygen species (respiratory burst), the release of granules filled with toxic enzymes (degranulation) and the NET formation (37). Further, neutrophils also secrete IL-17A that contributes to the dysregulated IL-17/IL-23 feedforward loop leading to a state of chronic inflammation (37, 38). In future experiments, a co-culture model with neutrophils could be used to mimic the effect of the neutrophils on the epidermis and closer analyze the interaction of keratinocytes and neutrophils in this context.

Strikingly, we observed in general a higher expression of S100A7 and a significantly increased secretion of IL-1α and IL-8 in the TLR3 KO epidermis models compared to the wild type (Figure 3C, upper panel, Figure 4B). Mechanistically these effects could be mediated by the transcription factor STAT3 as we observed an overall higher level of phosphorylated STAT3 in the TLR3 KO epidermis models (Figure 3C, right panel). Hyperactivation of STAT3 in keratinocytes is a characteristic of psoriatic skin (39) which was demonstrated once again in our recently developed *in vitro* psoriasis model based on STAT3 overexpressing keratinocytes (7). STAT3 is activated in keratinocytes in response to T cell secreted cytokines such as IL-22 or IL-6 but also indirectly by TLR3 simulation (15, 40). Our results of the wild type epidermis models, which showed an upregulation of phosphorylated STAT3 after stimulation with poly(I:C), confirm this. However, we also showed in two independent cell lines that the absence of TLR3 in keratinocytes induces inflammatory effects in our *in vitro* skin model (Figure 3C and Supplementary Figure 1). This indicates that the role of TLR3 in psoriasis is presumably more complex. Additional to skin inflammation, TLR3 plays a role in wound healing, skin regeneration, skin barrier maintenance and keratinocyte morphology and development (15, 41, 42). Due to this broad spectrum of different functions and complex signaling interactions, it seems reasonable that a KO of TLR3 in keratinocytes impairs other signaling pathways or induces up- or downregulation of other receptors and effector molecules potentially leading to the observed effects. Interestingly, it was previously shown in a mouse model, that TLR3 deletion (TLR3 ^−^/^−^) increased the level of secreted pro-inflammatory cytokines upon fungal infection of the lungs compared to the wild type (43). This indicates that in certain conditions TLR3 may exert a negative regulator function in the immune response. Anti-inflammatory effects of TLR are so far only described for TLR10 (44). To our knowledge, previous *in vitro* studies on the role of TLR3 in keratinocytes are mostly based on stimulation and overactivation of the receptor. The effect of TLR3 deletion in keratinocytes on a psoriasiform phenotype warrants further analysis.

Although the initial levels of psoriasis markers were clearly higher in TLR3 KO epidermis models compared to the wild type models, they did not significantly further increase after treatment with poly(I:C). Similar to the TLR2 data, we detected a significantly higher expression of IL-1α, IL-8 and CXCL-1 in wild type but not in the TLR3 KO epidermis models after stimulation with the respective TLR agonist (Figure 4B). We additionally measured elevated levels of psoriasis-associated proteins S100A9 and IL-23 after poly(I:C) stimulation of wild type epidermis models which supports the previous results from Kelemen et al. (20).

Thus, we demonstrated that both, TLR2 and TLR3 mediate the expression of psoriatic characteristics in normal *in vitro* epidermis models.

It has not escaped our notice that an increase in several of the analyzed immune mediators was also measured in the KO keratinocyte cell lines after stimulation with the agonists although this was in all cases significantly lower than in the wild type keratinocytes. As the knock-out efficiency was about 90%, there was still a minor number of TLR2 and TLR3 positive keratinocytes left in the models that could respond to the TLR signaling activation. It is also known, that poly(I:C) does not only activate the TLR3 signaling pathway but binds also to other cytosolic RNA receptors such as retinoic acid-inducible protein I (RIG-I) and melanoma differentiation-associate gene 5 (MDA-5) (45). Consequently, the upregulation of psoriasis markers, especially of S100A7 (Figure 3C, upper panel), in the TLR3 KO keratinocytes after stimulation with poly(I:C) could be mediated by RIG-I and MDA-5 (46).

Psoriasis is a multifaceted autoinflammatory disease that neither can be reduced to one biomarker nor to one signaling pathway. The combination of innate and adaptive immune responses mediates the complex disease phenotype. Nonetheless, taking together, the combination of the increase in S100A9, IL-23, IL-1α, CXCL-1 and IL-8 expression in keratinocytes indicates a psoriasiform-like skin inflammation. Thus, the activation of TLR signaling pathways by DAMPs and PAMPs like free nucleic acids or bacterial components in keratinocytes might be sufficient to trigger psoriatic reactions independent of immune cells.

The deeper understanding of signaling pathways in psoriasis and growing insights on the involved T cell subsets in the disease have led to the development of new targeted therapies. Novel biologics focus on psoriasis associated cytokines like TNF-α (e.g., adalimumab and infliximab) or the IL-23/IL-17 axis (e.g., secukinumab, ixekizumab) (47). Although biologics significantly improved the therapy efficacy, they still cannot prevent a relapse of the disease and their systemic application has been shown to cause adverse reactions (6). There are demands to complement the current T cell centric therapeutic approaches with drugs targeting early innate immunity events in keratinocytes (4, 6). In this work, we demonstrated a strong correlation between TLR2 and TLR3 activation in keratinocytes and expression of psoriasis markers. Keratinocyte expressed TLR2 and TLR3 offer an easy and druggable target, as TLR antagonists are small molecule inhibitors that could be administered topically on the skin. Further, they are less costly and could present a good alternative or addition to the current antibody therapies.

Summing up, we showed in *in vitro* epidermis models that the specific activation of TLR2 or TLR3 in keratinocytes induced the expression of psoriasis markers, especially of chemokines that are related to neutrophil recruitment. Thus, in our *in vitro* epidermis model the presence of TLR activators as described for the initiation phase of psoriasis, is sufficient to induce the expression of psoriasis associated molecules in keratinocytes without the involvement of immune cells.

## Data Availability

The original contributions presented in the study are included in the article/Supplementary material, further inquiries can be directed to the corresponding author.
