# Supplementary material for: Human epidermis models demonstrate mediator role of TLR2 and TLR3 for psoriatic inflammation
Source: Front Med (Lausanne). 2025 Sep 9;12:1663279. doi: 10.3389/fmed.2025.1663279 (PMC12456296; doi:10.3389/fmed.2025.1663279)
Supplement: Supplementary file 1 [file Data_Sheet_1.PDF]

Supplementary Material

1 Supplementary Figures

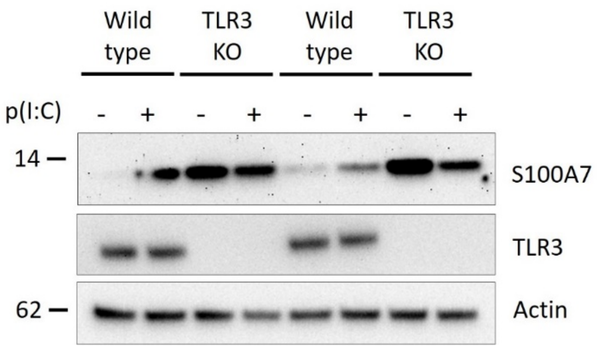

Suppl. Fig. 1: Stimulation of epidermis models set up from independently created TLR3 KO keratinocytes with poly(I:C). A second sgRNA that targets TLR3 exon 2 at another position (sgRNA 2, details see material and methods section) was used to create a second TLR3 KO keratinocyte cell line. 3D in vitro epidermis models were set up from wild type or TLR3 KO keratinocytes (sgRNA 2) and treated with 5 µg/mL poly (I:C) for 6 days during airlift culture. Expression of S100A7, TLR3 and actin in wild type and TLR3 KO epidermis models was analysed by Western Blot. Results are shown for two technical replicates.

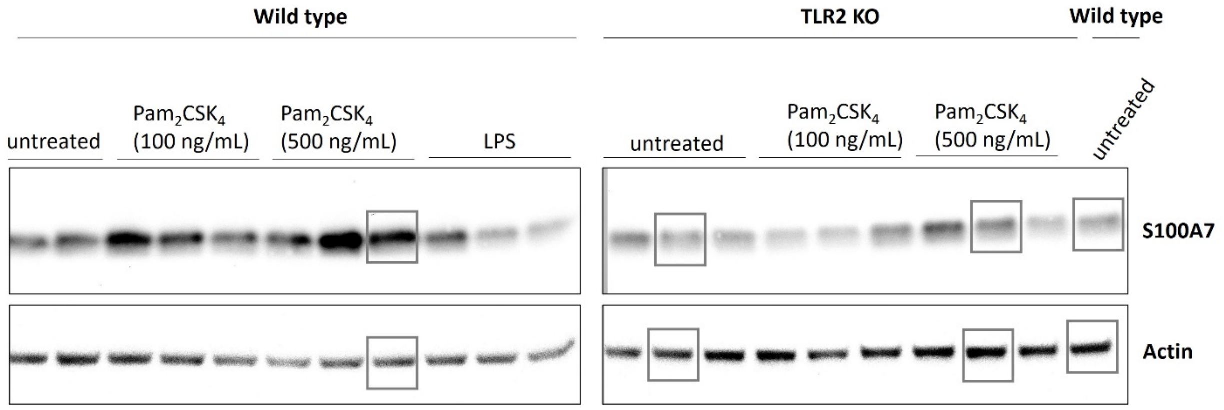

Suppl. Fig. 2: Representative full-length western blots of wild type and TLR2 KO epidermis models stimulated with Pam$_2$CSK$_4$. 3D in vitro epidermis models were set up from wild type or TLR2 KO keratinocytes and treated with Pam$_2$CSK$_4$ (100 and 500 ng/mL) or LPS (0,8 ng/mL) for 6 days during airlift culture. The grey squares indicate the sections shown in figure 2C.

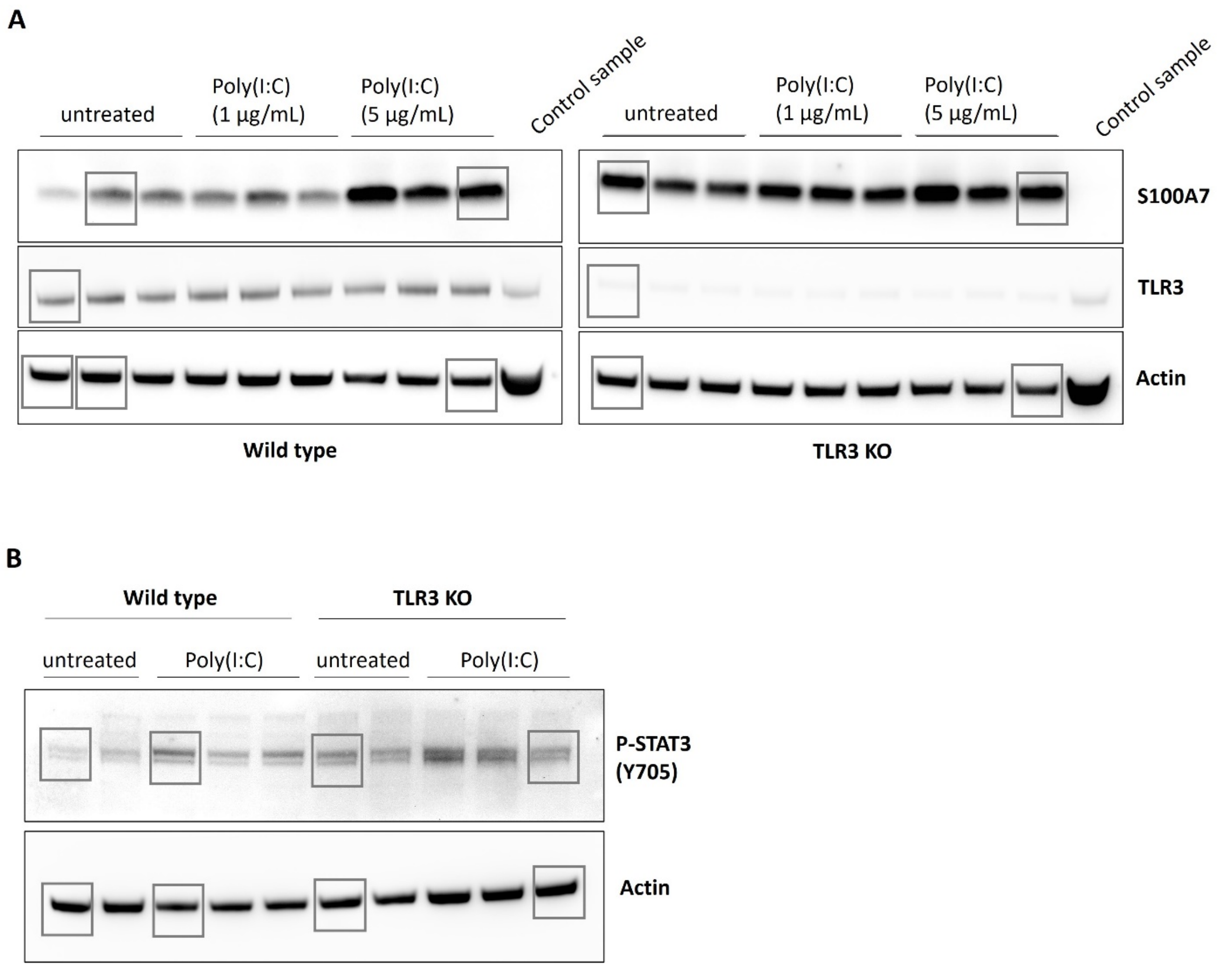

Suppl. Fig. 3: Representative full-length western blots of wild type and TLR3 KO epidermis models stimulated with poly(I:C). 3D in vitro epidermis models were set up from wild type or TLR3 KO keratinocytes and treated with poly(I:C) (1 µg/mL and 5 µg/mL) for 6 days during airlift culture. The grey squares indicate the sections shown in figure 3C left panel (A) and right panel (B). The control sample is a defined protein amount of a fibroblast cell lysate that was loaded on both gels.
